# Music performance anxiety, sleep problems, and disordered eating: the role of performance and learning self-efficacy

**DOI:** 10.3389/fpsyt.2026.1933259

**Published:** 2026-09-21

**Authors:** Shuai Jiao, Yaoyi Wu, Xuan Zhou

**Affiliations:** 1 Chang Zhi Medical College, Music Therapy Department, ChangZhi, China; 2 Performing Department, Communication University of Shanxi, Taiyuan, China; 3 School of Music and Dance, Xihua University, Chengdu, Sichuan, China

**Keywords:** disordered eating, music performance anxiety, music student, self-efficacy, sleep problems

## Abstract

**Background:**

Music students face chronic evaluative stress, increasing their vulnerability to sleep problems and disordered eating. Drawing on transdiagnostic and self-regulation frameworks, this study examined whether music performance anxiety (MPA) is associated with both outcomes and whether music performance and learning self-efficacy moderate these associations.

**Methods:**

A cross-sectional survey of 260 Chinese music students assessed music performance anxiety, sleep problems, disordered eating, and self-efficacy. Robust maximum likelihood structural equation modeling and Johnson–Neyman analyses were used, controlling body mass index, age, gender, and years of music study.

**Results:**

Music performance anxiety correlated with disordered eating (*r* = .24, *p* <.001) and sleep problems (*r* = .21, *p* <.001). In the structural model, music performance anxiety was positively associated with disordered eating (*β* = 0.25, *p* <.01). For sleep problems, music performance self-efficacy showed a negative main effect (β = −0.41, *p* <.001), whereas the direct effect of music performance anxiety was non-significant (β = 0.04, *p* >.05). The interaction between music performance anxiety and music performance self-efficacy was negatively associated with sleep problems (β = −0.41, *p* <.01). Johnson–Neyman analyses showed that music performance anxiety was associated with sleep problems only when music performance self-efficacy was < 3.80 or > 5.55.

**Conclusions:**

Music performance anxiety is a transdiagnostic risk factor among music students, directly relating to disordered eating, while its link to sleep problems depends on domain-specific self-efficacy. Interventions may combine anxiety reduction with balanced self-efficacy support to improve recovery under performance pressure.

## Background

International research has consistently documented a high prevalence of sleep problems and disordered eating behaviors among university students. Epidemiological surveys indicate that more than one third of college students report poor sleep quality or varying degrees of sleep disturbance, while irregular eating patterns, emotional eating, and impaired dietary control are likewise common in this population (1–3). A substantial body of research further suggests that sleep problems and disordered eating do not occur independently but frequently co-occur in contexts of heightened psychological stress and anxiety, and are closely associated with poorer mental health outcomes (4, 5).

From a transdiagnostic perspective, sleep problems and disordered eating can be conceptualized as distinct behavioral manifestations of a shared set of underlying mechanisms. Transdiagnostic models posit that a wide range of psychological and behavioral difficulties arise from common core processes—such as impaired emotion regulation, persistent cognitive rumination, and heightened physiological arousal—rather than from disorder-specific pathological pathways (6, 7). Within this framework, anxiety-related transdiagnostic mechanisms not only increase vulnerability to sleep disturbances but also substantially elevate the likelihood of disordered eating behaviors, thereby contributing to their frequent co-occurrence under high-stress conditions. This perspective provides an important theoretical rationale for examining sleep problems and eating-related problems simultaneously within a single analytical model. Beyond the acquisition of technical skills, active engagement in music has been associated with cognitive, social, and personal benefits, including attentional development, emotional expression, and self-esteem (8). Formal music training may also provide students with opportunities for artistic identity development and meaningful achievement. However, these potential benefits are often pursued within demanding practice schedules and highly evaluative performance settings, which may increase vulnerability to music performance anxiety among some students.

Among music students, this risk of comorbidity may be even more pronounced. Students in conservatories are typically required to engage in long-term, high-intensity practice and are frequently exposed to examinations, juried performances, and public evaluations, resulting in a learning environment characterized by intense competition and strong evaluative threat (5). Previous studies have shown that music students report significantly higher levels of anxiety and psychological distress than general university students, and are more likely to experience sleep deprivation and disrupted daily routines during periods of heightened performance pressure (9). In the Chinese context, music students similarly face rigorous professional training and high standards of evaluation. Recent evidence indicates that they exhibit generally elevated levels of music performance anxiety, which are significantly associated with lower subjective well-being (10). However, systematic investigations examining how music performance anxiety simultaneously contributes to sleep problems and eating-related problems among music students remain scarce. Accordingly, the present study focuses on the associations between music performance anxiety, sleep problems, and disordered eating in this population, and further explores the potential moderating role of self-efficacy.

### 1.1 Music performance anxiety and sleep problems

Music performance anxiety (MPA) refers to a persistent state of anxiety that arises in performance or performance-related evaluative contexts, and is characterized by concerns about negative evaluation, intense emotional tension, and pronounced physiological arousal (11, 12). From the perspective of the stress–arousal–self-regulation failure model, high levels of anxiety impair individuals’ capacity for self-regulation by inducing sustained physiological arousal and increasing cognitive load, thereby disrupting multiple health-related behavioral systems (13, 14).

Sleep is a behavioral process that relies heavily on effective self-regulation and the stability of physiological rhythms (15). A substantial body of research indicates that anxiety-related cognitive rumination and hyperarousal constitute core mechanisms underlying difficulties in sleep initiation, increased nocturnal awakenings, and reduced subjective sleep quality (4). In musical contexts, the heightened arousal associated with music performance anxiety is particularly pronounced during performance preparation and on the night before performances. Its effects often extend beyond the performance itself and may chronically disrupt students’ sleep–wake rhythms (11, 12). Although few studies have directly examined the association between music performance anxiety and sleep problems, a substantial body of research has consistently identified anxiety as a key factors of sleep problems (16, 17). Accordingly, music performance anxiety is likely to represent a key psychological factor of sleep problems among music students.

### 1.2 Music performance anxiety and disordered eating

Beyond sleep, disordered eating constitutes another important pathway through which anxiety influences individual health. International research has consistently shown that anxiety and psychological stress affect eating behavior via emotion regulation mechanisms, manifesting as emotional eating, dietary restraint, or disrupted eating rhythms (18). Among university students, higher levels of anxiety have been associated with irregular meal patterns, a preference for high-calorie foods, and decreased appetite (12). In musical contexts, music performance anxiety may influence disordered eating through multiple pathways. On the one hand, pre-performance tension and physiological discomfort (e.g., gastrointestinal distress and nausea) may directly suppress appetite. On the other hand, chronic psychological stress may lead some students to use eating as a means of emotion regulation, thereby fostering maladaptive eating patterns (11, 12).

Although sleep problems and disordered eating have often been examined separately in the existing literature, accumulating evidence indicates that these two health-related behaviors are closely intertwined under conditions of psychological stress. Insufficient sleep may exacerbate disordered eating through hormonal dysregulation and impaired cognitive control, whereas maladaptive eating behaviors may in turn disrupt sleep–wake rhythms, giving rise to a vicious cycle (4, 18).

In musical performance contexts, music performance anxiety may function as a common upstream stressor that simultaneously affects both sleep and eating systems. On the one hand, pre-performance tension and physiological discomfort (e.g., gastrointestinal distress and nausea) may directly suppress appetite. On the other hand, chronic anxiety may prompt some students to regulate their emotions through eating or lead to disrupted eating rhythms. Given the co-occurring nature of sleep problems and disordered eating, it is therefore necessary to systematically examine the effects of music performance anxiety on both health behaviors within a single analytical model.

### 1.3 The moderating role of music self-efficacy

Self-efficacy refers to individuals’ beliefs about their capability to organize and execute the actions required to manage prospective situations. Within social cognitive theory, self-efficacy is a central personal resource that shapes how people appraise demanding situations (e.g., as challenge vs. threat), how much effort they invest, how long they persist, and how effectively they regulate affect and behavior under pressure (19). In evaluative contexts such as public musical performance, higher self-efficacy is theorized to buffer the impact of performance-related stress by strengthening perceived control and coping capacity, thereby attenuating anxiety reactions and reducing the likelihood that stress responses disrupt attention, motor execution, and self-regulation (19).

In music education research, self-efficacy is typically conceptualized as domain- and task-specific. A common distinction is made between music performance self-efficacy and music learning self-efficacy. Performance self-efficacy reflects confidence in one’s capacity to perform successfully in public or evaluative settings, whereas learning self-efficacy reflects confidence in one’s capacity to acquire skills, refine technique, and improve through sustained practice and feedback (20, 21). Music performance self-efficacy refers to confidence in one’s ability to successfully perform in public or evaluative settings, whereas music learning self-efficacy emphasizes beliefs in one’s capacity to acquire skills, accumulate experience, and make continuous progress. Empirical evidence indicates that self-efficacy plays a significant role examining musical performance, practice behaviors, and stress coping, and is negatively associated with music performance anxiety (21, 22). Accordingly, students with higher levels of learning self-efficacy are more likely to remain goal-oriented during prolonged learning tasks and to plan their practice and daily schedules effectively, thereby maintaining more stable daily routines and healthier behavioral patterns when confronted with performance-related stress.

### 1.4 Current study

Taken together, although existing research has documented the prevalence of music performance anxiety and its detrimental effects on mental health, several important limitations remain. First, few studies have adopted a transdiagnostic perspective to simultaneously examine the effects of music performance anxiety on two co-occurring health outcomes—sleep problems and disordered eating—within a single analytical model. Second, limited attention has been paid to self-efficacy as a self-regulatory resource, and its potential moderating role in these associations has rarely been systematically tested. Third, empirical evidence remains particularly scarce among Chinese music students, a population that is chronically exposed to high levels of evaluative pressure.

Accordingly, the present study focuses on Chinese music students and, drawing on transdiagnostic and self-regulation frameworks, examines the associations of music performance anxiety on sleep problems and disordered eating. Furthermore, it examines the moderating roles of music performance self-efficacy and music learning self-efficacy in these relationships. Accordingly, we hypothesize that higher levels of music performance anxiety will be associated with a greater likelihood of sleep problems (H1) and disordered eating (H2) among students. Furthermore, we propose that music performance self-efficacy (H3) and music learning self-efficacy (H4) will buffer the adverse effects of music performance anxiety on both sleep problems and disordered eating (see **Figure 1**).

**Figure 1:**
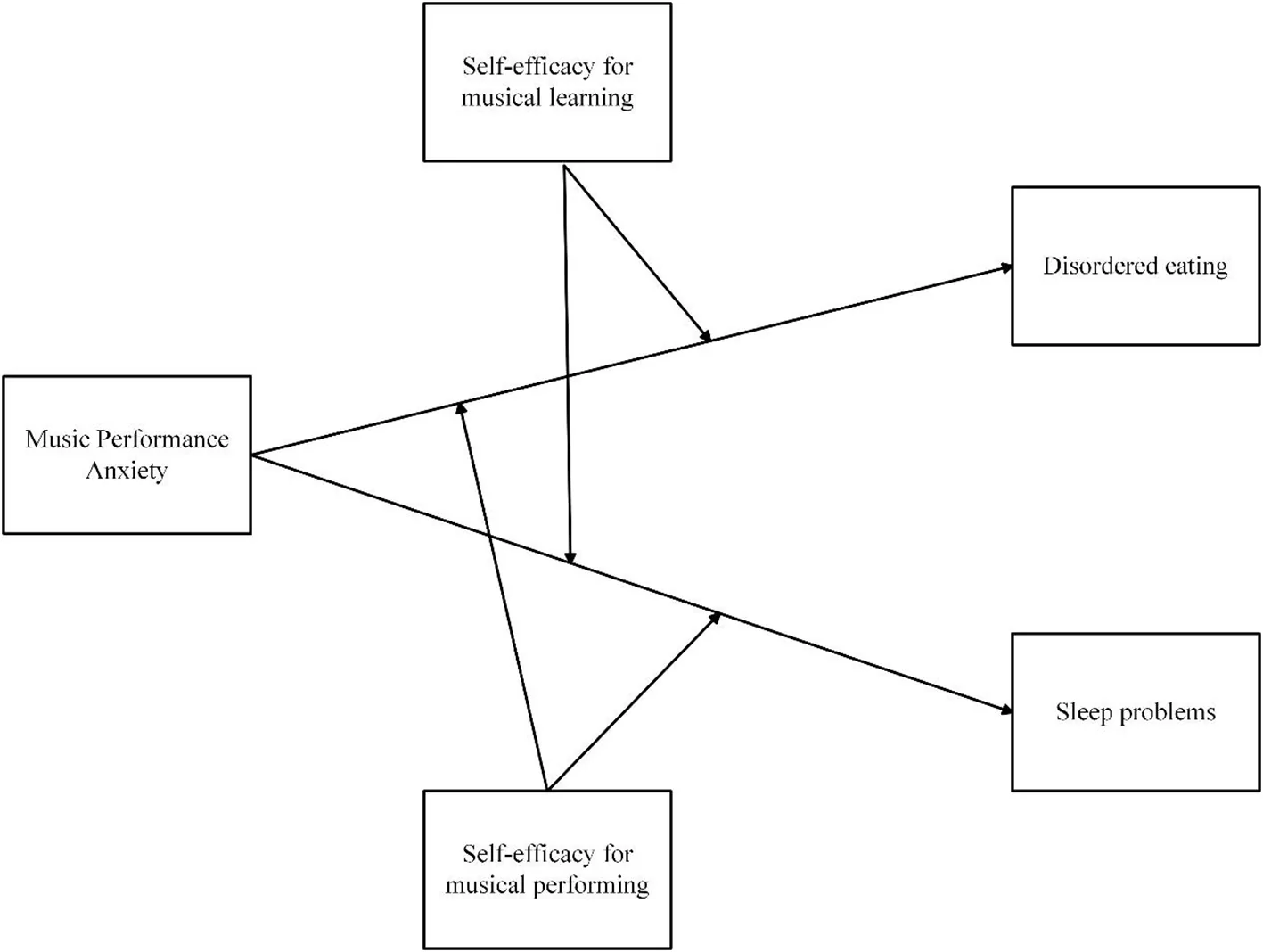
Conceptual model of the hypothesized relationships.

## 2 Methods

### 2.1 Participants and procedure

This study was approved by the Ethics Committee of Chang Zhi Medical College and conducted in accordance with the 1964 Helsinki Declaration and its subsequent amendments. Participants were music students receiving formal professional training, including students enrolled in music conservatories and university music programs, recruited from higher education institutions in Beijing, Tianjin, and Shijiazhuang, China.

Before participation, all respondents were presented with an online informed consent statement on the first page of the questionnaire, which described the study purpose, the voluntary and anonymous nature of participation, and the intended use of the data. Only individuals who provided informed consent were permitted to proceed with the survey. The survey was administered via the Wenjuanxing platform (www.wjx.cn), which restricted each IP address to a single submission. Recruitment was conducted through campus-based social media channels targeting music departments and conservatories; thus, a convenience sampling approach was used. A total of 299 participants initially completed the questionnaire; however, 39 responses were excluded due to failure on attention-check items (e.g., simple arithmetic questions such as “What is 3 + 4?”). The final analytic sample comprised 260 music students, including 94 men (36.2%) and 166 women (63.8%). Participants had a mean age of 19.37 years (SD = 1.17), a mean body mass index (BMI) of 21.03 kg/m² (SD = 3.88), and an average of 4.57 years of formal music training (SD = 2.95). Participants represented diverse music-related majors, most commonly music performance (n = 112, 43.1%) and music therapy (n = 55, 21.2%). Fifteen participants (5.8%) reported vocal studies, whereas 54 participants (20.8%) reported a specific instrumental specialization.

### 2.2 Measures

#### 2.2.1 Music performance anxiety scale

Music performance anxiety was assessed using the Mazzarolo Music Performance Anxiety Scale (M-MPAS; 23), a five-item self-report measure of music performance anxiety. The scale was developed to assess the intensity and frequency of performance-related anxiety, as well as aversion to or avoidance of future music performance. The final M-MPAS consists of five items, each rated on a 7-point Likert scale ranging from 0 (Strongly Disagree) to 6 (Strongly Agree). Higher scores indicate greater levels of music performance anxiety. The M-MPAS has also been used among Chinese music students (10). In the present study,Cronbach’s α is 0.71.

#### 2.2.2 Eating disorder examination questionnaire short

Disordered eating was assessed using the 12-item Eating Disorder Examination Questionnaire–Short Form (EDE-QS; 24). This self-report instrument employs a 4-point scale (0–3), with item scores summed to yield a total score ranging from 0 to 36, where higher scores indicate greater symptom severity. An example item asks whether an individual’s weight or shape influences their self-evaluation. The Chinese adaptation of the EDE-QS has demonstrated strong psychometric properties among young Chinese populations (25). In the present study, the scale exhibited good internal consistency (Cronbach’s α = 0.92).

#### 2.2.3 Sleep problems

Sleep problems were assessed using the Self-Rating Scale of Sleep (SRSS), a widely used self-report measure of subjective sleep quality in Chinese populations (26). The SRSS consists of 10 items assessing common sleep disturbances and sleep-related daytime dysfunction, including difficulties in sleep initiation, sleep maintenance, early morning awakening, insufficient sleep duration, and daytime fatigue. Participants rated the frequency of each symptom over a recent period on a 5-point Likert scale ranging from 1 (never) to 5 (always). Total scores range from 10 to 50, with higher scores indicating poorer sleep quality and more severe sleep problems. The SRSS has demonstrated good reliability and validity in prior studies involving Chinese adult and student samples, particularly in stress- and anxiety-related research contexts. In the present study, the internal consistency of the SRSS was acceptable (Cronbach’s α = 0.82).

#### 2.2.4 Music self-efficacy

This study utilized the version developed by Ritchie and Williamon (20), which comprises two subscales: the Music Learning Self-Efficacy Subscale (MLSE; 11 items) and the Music Performance Self-Efficacy Subscale (MPSE; 9 items). Sample items include: “I am confident that I can successfully learn the music for this performance” (MLSE) and “I am confident that I can succeed in the performance” (MPSE). Participants rated each item on a 7-point Likert scale, ranging from 1 (“Strongly Disagree”) to 7 (“Strongly Agree”). The original study reported Cronbach’s α values of 0.82 for MLSE and 0.78 for MPSE, indicating good internal consistency (20). In the present study, the Cronbach’s α values were 0.90 for MLSE and 0.89 for MPSE.

### 2.3 Data analysis

All analyses were conducted in R (version 4.1.3) using the lavaan package. As the online survey platform required responses to all items, no item-level missing data were present. Descriptive statistics and Pearson correlation analyses were first performed to examine bivariate associations among the study variables. Correlation effect sizes were interpreted following conventional guidelines, with values of r = .10,.30, and.50 representing small, medium, and large effects, respectively.

Structural equation modeling (SEM) was then employed to examine the hypothesized relationships among music performance anxiety, self-efficacy, and the two outcome variables. All study variables were modeled as observed scale scores. Before model estimation, continuous predictors were mean-centered. Two interaction terms were then created by multiplying the centered music performance anxiety score with the centered music performance self-efficacy score (MPAS × MPSE) and with the centered music learning self-efficacy score (MPAS × MLSE), respectively. The centered main-effect terms, the two interaction terms, and the covariates were entered simultaneously in the models examining sleep problems and disordered eating.

Models were estimated using robust maximum likelihood estimation (MLR) to account for potential deviations from multivariate normality. Standardized path coefficients, robust standard errors, and 95% confidence intervals were reported. To further probe significant interaction effects, Johnson–Neyman analyses were conducted to identify regions of significance across levels of the moderator. BMI, age, gender, and years of music study were included as covariates in all models.

## 3 Results

### 3.1 Correlations

Pearson correlation coefficients among MPAS, EDE-QS, SRSS, MLSE, and MPSE are presented in **Table 1**. MPAS was positively correlated with EDE-QS (r = .24, p <.001; small effect size) and SRSS (r = .21, p <.001; small effect size), and negatively correlated with MLSE (r = -.47, p <.001; large effect size) and MPSE (r = -.48, p <.001; large effect size). EDE-QS was positively correlated with SRSS (r = .22, p <.001; small effect size) but was not significantly correlated with MLSE (r = -.11, p = .07) or MPSE (r = -.11, p = .08). SRSS showed negative correlations with MLSE (r = -.43, p <.001; large effect size) and MPSE (r = -.46, p <.001; large effect size). A strong positive correlation was found between MLSE and MPSE (r = .79, p <.001; large effect size).

**Table 1:** Correlations of study variables.

Variable	MPAS	EDE-QS	SRSS	MLSE	MPSE
MPAS	1				
EDE-QS	.24***	1			
SRSS	.21***	.22***	1		
MLSE	-.47***	-0.11	-.43***	1	
MPSE	-.48***	-0.11	-.46***	.79***	1
*M* (SD)	14.38 (4.82)	11.29 (8.76)	24.43 (6.77)	4.79 (0.87)	4.61 (0.91)

MPAS, Music Performance Anxiety Scale; EDE-QS, Eating Disorder Examination Questionnaire–Short Form; SRSS, Self-Rating Scale of Sleep; MLSE, Music Learning Self-Efficacy; MPSE, Music Performance Self-Efficacy.

*p <.05; **p < .01; ***p < .001.

### 3.2 Moderation model

For disordered eating, music performance anxiety showed a significant positive association with disordered eating symptoms (β = 0.25, SE = 0.08, 95% CI [0.10, 0.40], p <.01), indicating that higher levels of performance anxiety were related to greater eating-related problems. Body mass index was also a significant was positively associated with disordered eating (β = 0.22, SE = 0.06, 95% CI [0.11, 0.34], p <.001). In contrast, neither music learning self-efficacy nor music performance self-efficacy showed significant main effects on disordered eating, and no significant interaction effects involving self-efficacy were observed (all ps >.05) (see **Table 2**).

**Table 2:** Moderation model results.

Variable	EDE-QS				SRSS			
	*β*	SE	95% CI (lower)	95% CI (upper)	*β*	SE	95% CI (lower)	95% CI (upper)
Gender	-0.02	0.06	-0.14	0.10	0.01	0.06	-0.10	0.12
BMI	0.22***	0.06	0.11	0.34	-0.02	0.06	-0.14	0.1
Age	0.02	0.06	-0.1	0.13	0.02	0.06	-0.09	0.13
Years of Music Study	-0.03	0.05	-0.13	0.07	-0.09	0.05	-0.19	0.02
MPAS	0.25**	0.08	0.10	0.40	0.04	0.06	-0.08	0.17
MLSE	0.02	0.1	-0.17	0.21	-0.13	0.1	-0.32	0.07
MPSE	0.06	0.1	-0.13	0.26	-0.41***	0.11	-0.62	-0.19
MPAS×MLSE	0.10	0.13	-0.15	0.36	0.24	0.12	-0.001	0.47
MPAS×MPSE	-0.05	0.13	-0.3	0.21	-0.41**	0.13	-0.66	-0.16

MPAS, Music Performance Anxiety Scale; EDE-QS, Eating Disorder Examination Questionnaire–Short Form; SRSS, Self-Rating Scale of Sleep; MLSE, Music Learning Self-Efficacy; MPSE, Music Performance Self-Efficacy.

*p < .05; **p < .01; ***p <.001.

For sleep problems, music performance self-efficacy demonstrated a significant negative main effect (β = −0.41, SE = 0.11, 95% CI [−0.62, −0.19], p <.001), suggesting that higher performance self-efficacy was associated with fewer sleep problems. The direct effect of music performance anxiety on sleep problems was not significant (β = 0.04, SE = 0.06, 95% CI [−0.08, 0.17], p >.05). However, a significant interaction between music performance anxiety and music performance self-efficacy emerged examining sleep problems (β = −0.41, SE = 0.13, 95% CI [−0.66, −0.16], p <.01), indicating that the association between music performance anxiety and sleep problems varied as a function of music performance self-efficacy. In addition, the interaction between music performance anxiety and music learning self-efficacy showed a marginally positive effect on sleep problems (β = 0.24, SE = 0.12, 95% CI [−0.001, 0.47], p = .054), suggesting a trend whereby the relationship between music performance anxiety and sleep problems varied as a function of learning self-efficacy. Overall, these results indicate that whereas music performance anxiety is directly associated with disordered eating, its association with sleep problems appears to be conditional on self-efficacy resources, particularly music performance self-efficacy (see **Figure 2**).

**Figure 2:**
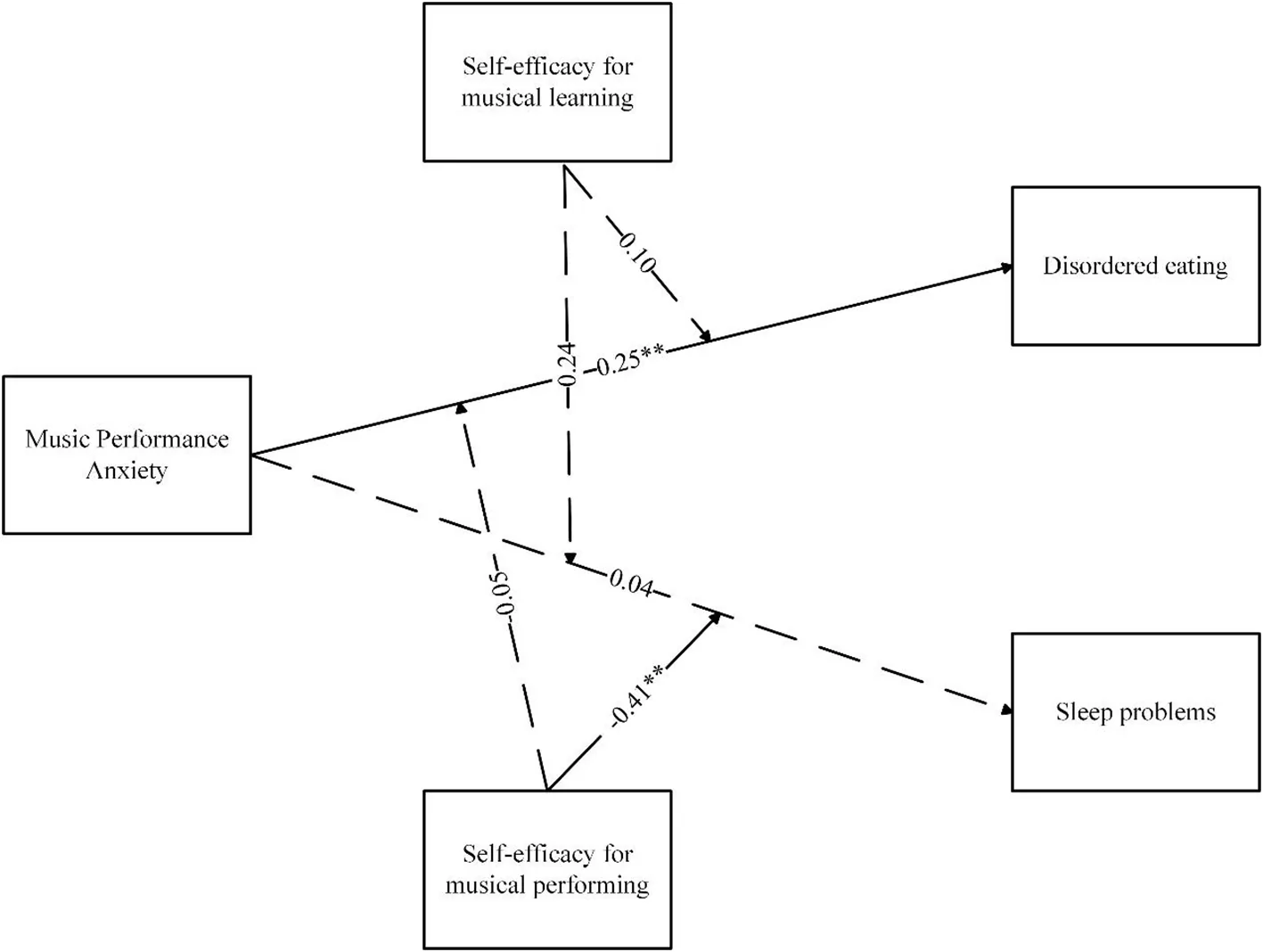
Moderation model results. *p < .05; **p < .01; ***p < .001.

Johnson–Neyman analyses were conducted to further probe the interaction between music performance anxiety and music performance self-efficacy examining sleep problems. The observed range of music performance self-efficacy in the sample was 1.67 to 7.00. The results indicated that when music performance self-efficacy fell between 3.80 and 5.55, the association between music performance anxiety and sleep problems was not statistically significant. In contrast, when music performance self-efficacy was below 3.80 or above 5.55, music performance anxiety significantly associated with sleep problems (p <.05). These findings suggest that the relationship between music performance anxiety and sleep problems is conditional on levels of performance self-efficacy and is primarily evident among students with relatively low or high self-efficacy, whereas the association is attenuated at moderate levels of self-efficacy (see **Figure 3**).

**Figure 3:**
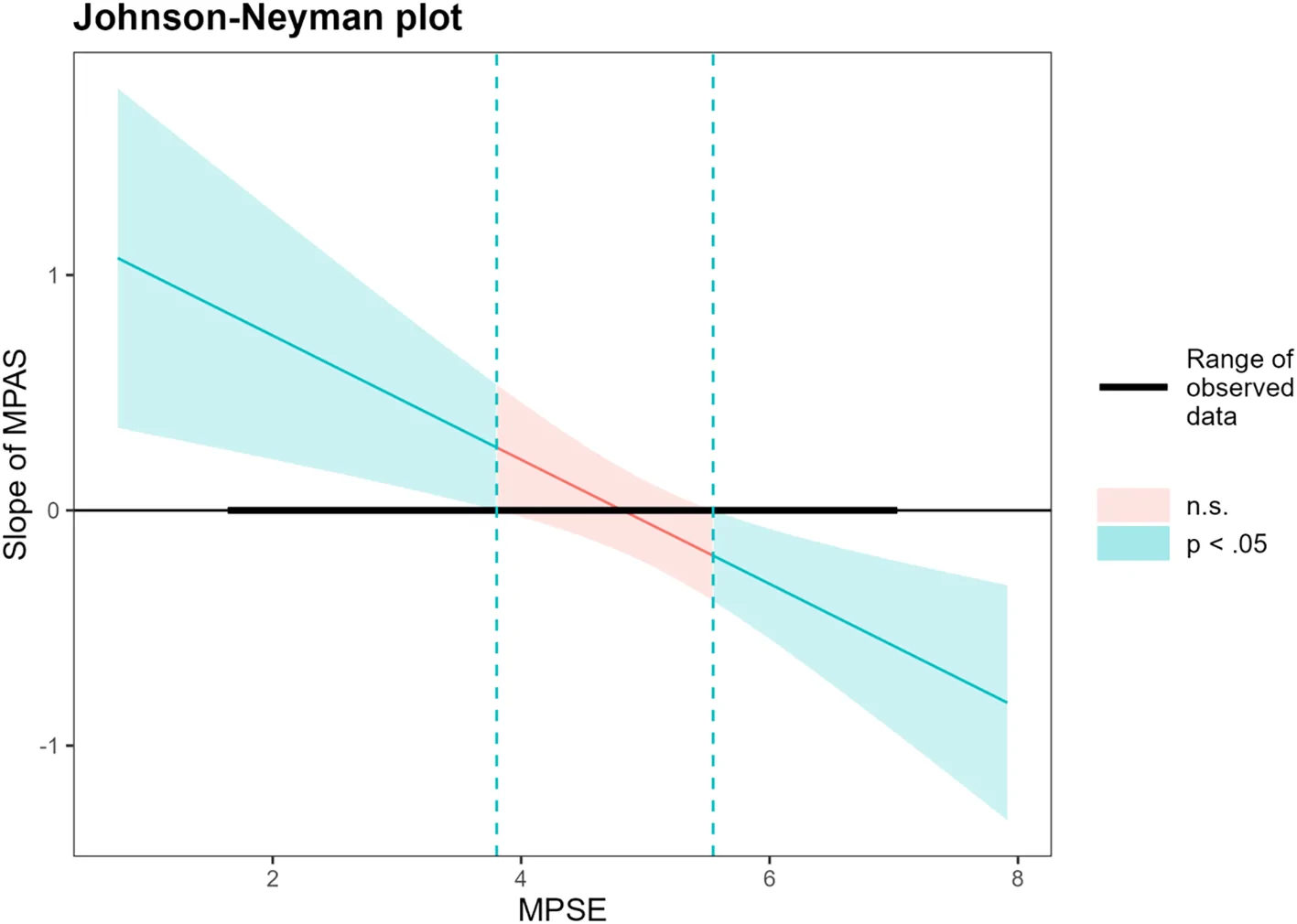
Johnson–Neyman plot of the association between music performance anxiety and sleep problems across levels of music performance self-efficacy. MPAS, Music Performance Anxiety Scale; MPSE, Music Performance Self-Efficacy.

## 4 Discussion

Drawing on transdiagnostic and self-regulation frameworks, the present study examined how music performance anxiety relates to sleep problems and disordered eating among Chinese music students, and whether music performance self-efficacy and music learning self-efficacy moderate these associations. Overall, the results provide partial support for the proposed hypotheses and reveal a differentiated pattern across health outcomes. Specifically, music performance anxiety was directly associated with greater disordered eating symptoms, whereas its association with sleep problems was conditional on students’ self-efficacy resources rather than reflecting a simple main effect. In addition, music performance self-efficacy showed a strong protective association with sleep problems. These findings suggest that music performance anxiety constitutes a salient psychological risk factor for health-related behavioral dysregulation among music students, but its effects vary across behavioral domains and depend on the configuration of domain-specific self-efficacy resources.

### 4.1 Music performance anxiety and sleep problems (H1)

Hypothesis 1 proposed that higher levels of music performance anxiety would be associated with more severe sleep problems. This hypothesis was partially supported. Music performance anxiety was positively correlated with sleep problems at the bivariate level; however, its direct effect became non-significant in the structural model after accounting for self-efficacy and interaction terms. This pattern suggests that the relationship between music performance anxiety and sleep problems is not uniform but depends on individual differences in self-regulatory resources.

This finding is consistent with prior research indicating that anxiety-related sleep disturbances are strongly shaped by cognitive and behavioral moderators rather than anxiety intensity alone (4, 27). Anxiety has been associated with sleep problems sleep problems primarily through heightened physiological arousal and persistent cognitive rumination, which interfere with sleep initiation and maintenance (4, 28). However, whether anxiety translates into actual sleep disruption depends on individuals’ capacity to downregulate arousal and disengage from threat-related thoughts before sleep.

In musical performance contexts, these demands are particularly pronounced. Performance anxiety often peaks during performance preparation and immediately before evaluations, periods in which students are required to balance high arousal with effective recovery (11, 12). The present findings suggest that music performance anxiety alone may be insufficient to predict sleep problems unless accompanied by insufficient or maladaptive self-regulatory resources, highlighting the importance of examining conditional effects rather than relying solely on main-effect models.

### 4.2 Music performance anxiety and disordered eating (H2)

In contrast to the conditional pattern observed for sleep problems, Hypothesis 2 was clearly supported: music performance anxiety showed a significant direct association with disordered eating symptoms, even after controlling for BMI and demographic variables. This finding aligns with a large body of literature demonstrating that anxiety and chronic psychological stress are robust predictors of maladaptive eating behaviors (18, 29).

From a transdiagnostic perspective, anxiety may disrupt eating behavior through impaired emotion regulation, increased physiological arousal, and altered interoceptive sensitivity (6). In the context of musical performance, anxiety-related somatic symptoms—such as gastrointestinal discomfort, nausea, or appetite suppression—are common and may directly interfere with normal eating, particularly around performance periods (11). At the same time, chronic exposure to evaluative stress may lead some students to use eating as a compensatory strategy to regulate negative affect, thereby increasing vulnerability to disordered eating patterns over time.

Notably, neither music performance self-efficacy nor music learning self-efficacy moderated the association between music performance anxiety and disordered eating. This suggests that eating-related dysregulation may be less sensitive to task-specific confidence and more strongly driven by affective and physiological processes. Similar patterns have been observed in non-musical samples, where anxiety–eating associations persist even after accounting for perceived control or coping beliefs (30, 31). Thus, disordered eating may represent a more direct and less cognitively buffered pathway through which music performance anxiety affects health.

### 4.3 The moderating role of music performance self-efficacy (H3)

Hypothesis 3 proposed that music performance self-efficacy would buffer the adverse effects of music performance anxiety on sleep problems and disordered eating. The results provided partial support for this hypothesis. Music performance self-efficacy showed a strong negative main effect on sleep problems, indicating that students with higher confidence in their performance ability reported better sleep quality. However, its moderating role was specific to sleep outcomes and did not extend to disordered eating.

This finding is consistent with social cognitive theory, which emphasizes self-efficacy as a key personal resource for regulating stress responses in evaluative situations (19, 32). Students with higher performance self-efficacy are more likely to appraise performance demands as manageable challenges rather than uncontrollable threats, thereby reducing anticipatory anxiety, physiological hyperarousal, and pre-sleep cognitive rumination—processes that are central to anxiety-related sleep disturbance (7, 33).

Importantly, the significant MPAS × MPSE interaction indicated that music performance self-efficacy meaningfully qualified the association between music performance anxiety and sleep problems. The Johnson–Neyman analysis showed that, at relatively low levels of music performance self-efficacy, higher music performance anxiety was associated with more sleep problems. At relatively high levels of performance self-efficacy, however, this association became negative, whereas no significant association was observed at moderate levels of performance self-efficacy. Rather than indicating a simple buffering effect, this crossover interaction suggests that the association between music performance anxiety and sleep problems varies depending on students’ level of performance-related confidence. One possible interpretation is that, among students with low-performance self-efficacy, anxiety may reflect perceived lack of control and heightened threat, thereby contributing to greater cognitive and physiological arousal and poorer sleep. In contrast, among students with high performance self-efficacy, greater performance anxiety may coexist with stronger perceived competence and more effective coping or preparation, such that anxiety does not necessarily translate into greater sleep disturbance. Because the present study is cross-sectional, however, the mechanisms underlying this pattern should be examined directly in future longitudinal or experimental research.

The absence of moderation effects for disordered eating further suggests that eating-related dysregulation may be less sensitive to performance-specific confidence and more directly driven by affective and physiological stress responses. Together, these findings suggest that the moderating role of music performance self-efficacy may be outcome-specific and that its association with anxiety-related sleep problems is more complex than a uniform buffering effect.

### 4.4 The moderating role of music learning self-efficacy (H4)

Hypothesis 4 examined whether music learning self-efficacy would moderate the associations between music performance anxiety and health outcomes. The findings provided only limited support for this hypothesis. Music learning self-efficacy did not show a significant direct association with sleep problems or disordered eating, nor did it significantly moderate the association between music performance anxiety and disordered eating. Although a marginal MPAS × MLSE interaction emerged in the model of sleep problems examining sleep problem (β = 0.24, p = .054), this effect did not reach the conventional threshold for statistical significance and its positive direction did not support the hypothesized buffering role of learning self-efficacy. This finding should therefore be interpreted cautiously and warrants replication in future research.

These findings suggest that learning-related confidence may play a more limited role in shaping the health correlates of music performance anxiety than performance-specific self-efficacy. This distinction may reflect the domain specificity of self-efficacy beliefs: confidence in one’s capacity to learn and improve may not directly translate into confidence in managing the immediate evaluative demands of public performance. Future research should further distinguish these forms of self-efficacy and examine whether they become more consequential under different learning, practice, and performance contexts. More broadly, recent meta-analytic evidence indicates that mindfulness- and acceptance-based interventions are associated with reductions in music performance anxiety among musicians (34). Such approaches may provide a useful direction for future research examining how anxiety management can be integrated with support for adaptive learning-related confidence and recovery among music students.

### 4.5 Limitations and future directions

Several limitations of the present study should be acknowledged. First, the cross-sectional design precludes causal inference regarding the directionality of the observed associations. Although the theoretical framework assumes that music performance anxiety contributes to sleep problems and disordered eating, reciprocal or bidirectional relationships are also plausible. Future longitudinal or experimental studies are needed to clarify temporal dynamics and causal mechanisms. Second, all variables were assessed using self-report measures, which may introduce common method bias. Future research could incorporate multi-method approaches, such as objective sleep indicators, ecological momentary assessment of anxiety, or clinician-rated measures of eating pathology, to strengthen measurement validity. Third, the sample consisted of Chinese music students, which enhances contextual specificity but may limit generalizability. Replication across different cultural contexts and musical training environments would help determine the robustness of the findings.

## 5 Conclusion

The present study provides evidence that music performance anxiety was significantly associated with health-related behavioral difficulties among Chinese music students. From a transdiagnostic perspective, performance anxiety was directly associated with disordered eating, whereas its association with sleep problems depended on domain-specific self-efficacy resources. Music performance and learning self-efficacy played distinct and interacting roles in shaping how anxiety-related processes translated into sleep disturbance, underscoring the importance of differentiating multiple forms of self-efficacy in high-evaluative contexts. These results suggest that interventions for music students should move beyond anxiety reduction alone and incorporate strategies that foster balanced self-efficacy and effective recovery from performance-related stress.

## Data Availability

The raw data supporting the conclusions of this article will be made available by the authors, without undue reservation.
